# *BRAF-V600* Mutations Have No Prognostic Impact in Stage IV Melanoma Patients Treated with Monochemotherapy

**DOI:** 10.1371/journal.pone.0089218

**Published:** 2014-02-20

**Authors:** Diana Meckbach, Ulrike Keim, Sabina Richter, Ulrike Leiter, Thomas K. Eigentler, Jürgen Bauer, Annette Pflugfelder, Petra Büttner, Claus Garbe, Benjamin Weide

**Affiliations:** 1 Division of Dermatooncology, Department of Dermatology, University Medical Center Tübingen, Tübingen, Germany; 2 Skin Cancer Research Group, School of Public Health, Tropical Medicine and Rehabilitation Sciences, James Cook University, Townsville, Australia; 3 German Cancer Research Center (DKFZ), Heidelberg, Germany; 4 German Cancer Consortium (DKTK), Heidelberg, Germany; University of Queensland Diamantina Institute, Australia

## Abstract

**Background:**

The impact of *BRAF* tumor mutations on the natural course of disease of melanoma patients is controversial.

**Patients and Methods:**

We analyzed the mutational status and overall survival of 215 patients receiving treatment with dacarbazine or temozolomide. All patients who started first-line treatment at our institution between 2000 and 2010 were included to prevent selection and bias due to thereafter arising therapeutic options.

**Results:**

No patient received *BRAF*- or *MEK-*inhibitors during follow-up. Survival was associated with the pattern of visceral involvement, the presence of brain metastases and the serum lactate dehydrogenase level (all p<0.001). The *BRAF-V600* mutational status was not associated with survival and no differences in overall survival were detected according to age, gender or to the cytotoxic agent used for therapy. In Cox regression analysis the presence of brain metastases (hazard ratio 2.3; p<0.001) and an elevated serum LDH (hazard ratio 2.5; p<0.001) were the only factors, which independently predicted survival.

**Conclusions:**

No differences in prognosis were observed according to the *BRAF* mutational status in patients with distant metastasis treated with monochemotherapy.

## Introduction

*BRAF-V600* tumor mutations constitutively activate the mitogen-activated protein kinase (MAPK) signaling pathway leading to an enhanced mitotic activity [1], [2]. Blocking the activated pathway by specific inhibitors leads to impressive clinical responses and an improved survival of advanced melanoma patients [3]–[5]. Nevertheless, the prognostic relevance of *BRAF* mutations in the natural course of disease is controversial [6]–[18]. A trend towards worse survival of metastatic patients with *BRAF* mutation was observed in two Australian cohorts and in a study performed in the United States [7]–[9]. Similarly, a poorer survival of metastatic patients with *BRAF* or *NRAS* tumor mutations [10] and of patients with *BRAF*-mutant tumors after treatment with temozolomide and bevacizumab [11] was reported before. In contrast, Edlundh-Rose *et al.* did not find any association between the tumor *NRAS* or *BRAF* genotype and survival after occurrence of metastasis [12].

The aim of the present study was to investigate the prognostic impact of *BRAF-V600* tumor mutations in melanoma patients receiving first-line treatment with dacarbazine or temozolomide during the years 2000–2010, before availability of *BRAF* inhibitors. We present the first survival analysis of melanoma patients focusing on the *BRAF* mutational status of an unselected real life cohort.

## Materials and Methods

### Ethics Statement

All patients had given their written informed consent to have clinical data recorded by the Central Malignant Melanoma Registry (CMMR) registry. The institutional ethics committee Tübingen approved the study (ethic vote 047/2013BO2).

### Patients

Patients with invasive cutaneous melanoma treated at the University Department of Dermatology Tübingen (Germany) were identified in the Central Malignant Melanoma Registry (CMMR) database. Of 319 patients who received first-line systemic treatment with dacarbazine or temozolomide between 2000 and 2010, formalin-fixed paraffin-embedded tumor tissue was available in 219 patients. Data obtained for each patient included gender, age, site of distant metastasis according to the American Joint Committee on Cancer (soft tissue metastasis vs. pulmonary involvement vs. other visceral sites), presence of brain metastasis, serum LDH level (normal vs. >upper limit of normal [ULN]) and the date and cause of death, if applicable. Moreover, time points of initiation of first-line chemotherapy and last follow-up were collected. All patients had given their written informed consent to have their data recorded by the CMMR. The aims and methods of data collection by the CMMR have previously been reported in detail [19].

### Sequencing

Microdissection of formalin-fixed paraffin-embedded tumor tissue was performed to obtain at least 50% tumor cells. After digestion by proteinase K an amplicon containing the *BRAF* codon 600 was amplified by a polymerase-chain-reaction (PCR) assay using forward primer 5′-tcataatgcttgctctgatagga-3′ and reverse primer 5′-ccaaaaatttaatcagtgga-3′. PCR products were analyzed on an agarose gel and purified using USB® ExoSAP-IT® (Affymetrix, Santa Clara, CA). Sanger sequencing was performed in reverse direction and sequences were analyzed with Mutation Surveyor Version 3.20 (SoftGenetics, State College, PA). For all samples which could not be clearly classified as mutant or wild-type, PCR and sequencing was repeated.

### Statistics

Overall survival time was calculated from the first application of temozolomide or dacarbazine to the date of last follow-up or death; only deaths due to melanoma were considered, whereas patients who died from other causes were censored at the date of death. Estimates of cumulative survival probabilities according to Kaplan-Meier were described together with 95%-confidence intervals and compared using log rank tests. Cox regression analyses were used to determine the independent effects of prognostic factors. All variables were considered in Cox regression analyses and patients with missing data were excluded. Models were established using backward and forward stepwise procedures. Remaining non-significant factors were assessed for potential confounding effects. Changes in the estimates of factors in a model by more than 5% were taken as indicative for confounding. Results of the Cox regression models were described by hazard ratios (HR) together with 95%-confidence intervals, and p-values were based on the Wald test. All Chi square tests were performed 2-sided using Fisher’s exact tests. Throughout the analysis, p-values of less than 0.05 were considered statistically significant. All analyses were carried out using SPSS Version 21 (IBM SPSS, Chicago, Illinois, USA).

## Results

### Patients

215 of 219 patients with successful sequencing (98.2%) were further analyzed. Median age was 64 years and 55% were male. The majority of patients (66.0%) were classified as M1c stage according to AJCC at start of systemic treatment (24.2% M1b and 9.8% M1a, respectively). During follow-up, 191 (88.8%) died from melanoma. Median follow-up was 9 months for patients who died and 46 months for those who were alive at the last follow-up. None of the patients received treatment with *BRAF* or *MEK* inhibitors during follow-up. A *BRAF-V600* tumor mutation was detected in 89 patients (41.4%). 80.9% of mutations were V600E, 18.0% and 1.1% were V600K and V600M mutations, respectively.

After stratification according to the *BRAF* mutational status, a comparison of both groups was performed to detect imbalanced distribution of the remaining factors (Table 1). Significant differences were found according to age with a higher proportion of younger patients in the tumor *BRAF* mutant compared to the *BRAF* wild-type group (31.5% vs. 15.1% younger than 50 years, respectively). Moreover, the proportion of patients treated with temozolomide was higher in the *BRAF* mutant group (43.8% vs. 26.2%). Both groups were well balanced for the remaining factors.

**Table 1:** Clinical characteristics and survival analysis.

	n	%	Mutational rate	% within *BRAF-V600* mutant patients	% within *BRAF-V600* wild-type	p*	1-year survival rate	[95%-CI#] (%)	p^$^
**All patients**	215	100%	41.4%				43.5%	[36.8; 50.2]	
***BRAF-V600*** ** mutations**									0.966
Present	89	41.4%					43.5%	[33.1; 53.9]	
Absent	126	58.6%					43.5%	[34.7; 52.3]	
**Gender**						0.071			0.071
Male	119	55.3%	47.1%	62.9%	50.0%		39.2%	[30.4; 48.0]	
Female	96	44.7%	34.4%	37.1%	50.0%		48.9%	[38.7; 59.0]	
**Age**						0.004			0.938
<50 years	47	21.9%	59.6%	31.5%	15.1%		39.7%	[25.5; 53.8]	
50–59 years	41	19.1%	51.2%	23.6%	15.9%		45.4%	[30.0; 60.8]	
60–69 years	52	24.2%	34.6%	20.2%	27.0%		45.2%	[31.5; 58.9]	
≥70 years	75	34.9%	29.3%	24.7%	42.1%		43.6%	[32.3; 54.9]	
**Systemic treatment**						0.008			0.146
Dacarbazine	143	66.5%	35.0%	56.2%	73.8%		46.3%	[38.1; 54.5]	
Temozolomide	72	33.5%	54.2%	43.8%	26.2%		37.8%	[26.4; 49.2]	
**LDH**						0.642			<0.001
Elevated	63	32.5%	38.1%	30.4%	33.9%		23.7%	[12.9; 34.4]	
Normal	131	67.5%	42.0%	69.6%	66.1%		49.1%	[40.5; 57.7]	
Missing	21								
**Brain metastasis**						0.307			<0.001
Yes	45	20.9%	48.9%	24.7%	18.3%		18.7%	[7.1; 30.3]	
No	170	79.1%	39.4%	75.3%	81.7%		50.0%	[42.4; 57.6]	
**Site of distant metastasis**						0.128			<0.001
Soft tissue	28	13.0%	35.7%	11.2%	14.3%		59.5%	[41.0; 78.0]	
Only lung	59	27.4%	52.5%	34.8%	22.2%		49.2%	[36.4; 61.9]	
Other visceral	128	59.5%	37.5%	53.9%	63.5%		37.4%	[29.0; 45.8]	

# 95%-CI = 95% confidence interval;

*p-values are results of Fishers exact tests $ p-values are results of log rank tests excluding cases with missing values.

Additionally, correlations were observed between the treatment with temozolomide and younger age (p = 0.001) and between treatment with temozolomide and the presence of brain metastases (p<0.001).

### Survival Analysis

**Figure 1:**
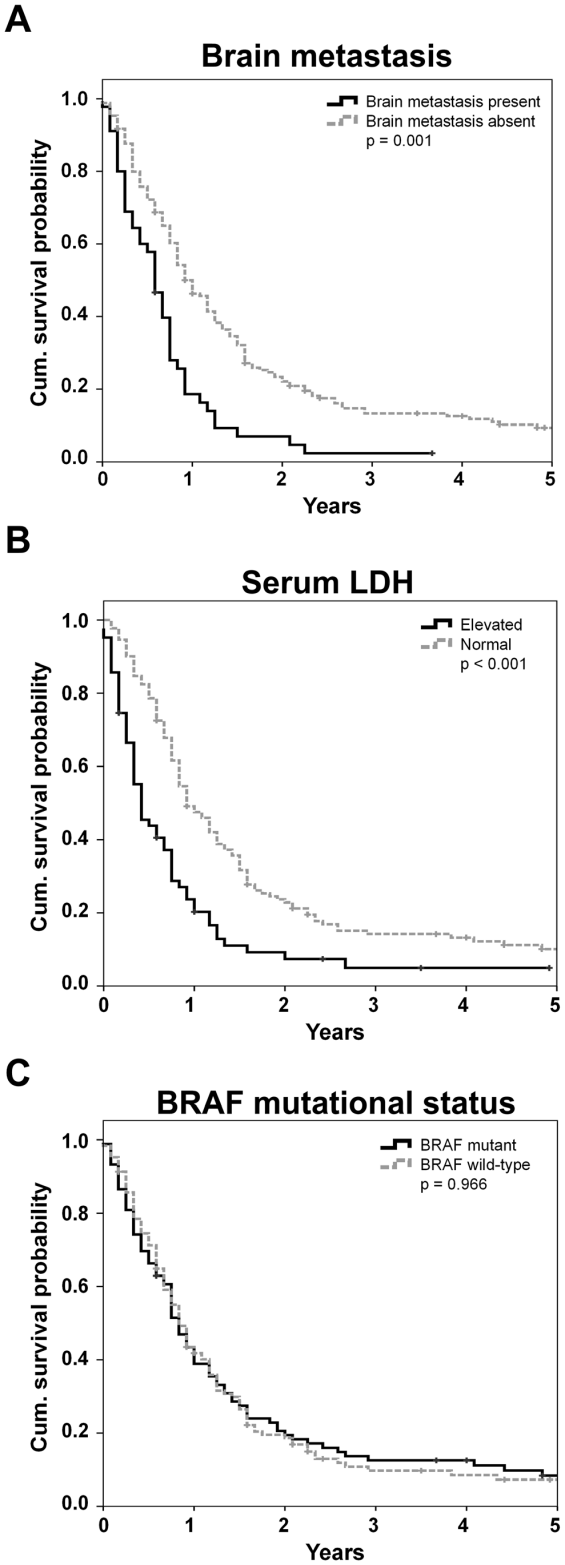
Univariate survival analysis. Kaplan Meier survival curves according to (A) the presence of brain metastasis, (B) serum lactate dehydrogenase (LDH) or (C) the *BRAF-V600* mutational status. Censored events are indicated by vertical lines.

Median overall survival probability according to Kaplan-Meier was 9 months. The presence of brain metastases (Figure 1A), the M category according to AJCC and the serum LDH level (Figure 1B) were associated with outcome (all p<0.001). The largest difference in the one year survival was observed according to cerebral involvement. Moreover, the presence of brain metastases was associated with the lowest absolute 1 year survival rate (18.1%). The best prognosis with a 59.5% 1-year survival rate was observed in patients with distant metastasis limited to the soft-tissue. The *BRAF-V600* mutational status was not associated with survival (p = 0.966; Figure 1C) and no differences in overall survival were detected according to age (p = 0.938), gender (p = 0.071) or to the cytotoxic agent used for therapy (dacarbazine vs. temozolomide; p = 0.146). Complete results of clinical associations and univariate survival analysis are presented in Table 1.

In Cox regression analysis (Table 2) the presence of brain metastases (HR 2.3; p<0.001) and an elevated serum LDH (HR 2.5; p<0.001) were the only factors, which independently predicted survival. No prognostic relevance was observed according to the *BRAF* mutational status.

**Table 2:** Multivariate analysis for disease-specific death.

Prognostic factor	Sample size (n = 194)	% Dead	Relative risk (95%-CI#)	p-value
Brain metastasis				
No	153 (78.9%)	86.3%	1	
Yes	41 (21.1%)	95.1%	2.5 (1.6, 3.8)	P<0.001
Lactate Dehydrogenase				
Normal	63 (32.5%)	87.0%	1	
Elevated	131 (67.5%)	90.5%	2.3 (1.6, 3.2)	P<0.001

# 95%-CI = 95% confidence interval;

*21 patients had unknown values for LDH and were excluded; the model was adjusted for the confounding effects of the site of distant metastasis; no significant interaction was detected.

Due to the observed correlations, we additionally performed the survival analysis separately for 170 patients without brain involvement (Table 3) and for the other 45 patients with brain metastasis (Table 4).

**Table 3:** Survival analysis for patients without brain involvement.

				Univariate Analysis			Multivariate Analysis^*^		
	n	%	% dead	1-year survival rate	[95%-CI^#^] (%)	p	Relative risk	[95%-CI^#^]	p
**All patients**	170	53.8	87.1	50.0	[42.4; 57.6]				
***BRAF-V600*** ** mutations**						0.714			
Present	67	39.4	91.0	52.2	[40.2; 64.2]				
Absent	103	60.6	84.5	48.5	[38.7; 58.3]				
**Gender**						0.123			
Male	92	54.1	90.2	45.3	[35.1; 55.5]				
Female	78	45.9	83.3	55.6	[44.4; 66.8]				
**Age**						0.878			
<50 years	33	19.4	90.9	48.5	[31.4; 65.6]				
50–59 years	32	18.8	84.4	52.1	[34.5; 69.7]				
60–69 years	41	24.1	87.8	47.6	[32.1; 63.1]				
≥70 years	64	37.6	85.9	51.2	[38.9; 63.5]				
**Systemic treatment**						0.962			
Dacarbazine	132	77.6	85.6	49.4	[40.8; 58.0]				
Temozolomide	38	22.4	92.1	51.9	[35.8; 68.0]				
**LDH**						<0.001			
Elevated	49	32.0	89.8	29.3	[16.4; 42.2]		2.3	[1.6; 3.4]	<0.001
Normal	104	68.0	84.6	56.2	[46.6; 65.8]		1.0		
Missing	17								
**Site of distant metastasis**						0.161			
Soft tissue	28	16.5	82.1	59.5	[41.1; 77.9]				
Only lung	59	34.7	96.6	49.2	[36.5; 61.9]				
Other visceral	83	48.8	81.9	47.4	[36.6; 58.2]				

# 95%-CI = 95% confidence interval; * 17 patients had unknown values for LDH and were excluded; the model was adjusted for the confounding effects of the site of distant metastasis; no significant interaction was detected.

**Table 4:** Survival analysis for patients with brain involvement.

				Univariate Analysis			Multivariate Analysis*		
	n	%	% dead	1-year survival rate	[95%-CI#] (%)	p	Relative risk	[95%-CI#]	p
**All patients**	45	100.0	95.6	18.7	[7.1; 30.3]				
***BRAF-V600*** ** mutations**						0.575			
Present	22	48.9	90.9	15.6	[0.0; 31.5]				
Absent	23	51.1	100.0	21.7	[4.8; 38.6]				
**Gender**						0.344			
Male	27	60.0	96.3	18.5	[3.8; 33.2]				
Female	18	40.0	94.4	18.3	[0.0; 36.7]				
**Age**						0.373			
<50 years	14	31.1	92.9	17.8	[0.0; 37.6]				
50–59 years	9	20.0	88.9	22.2	[0.0; 49.4]				
60–69 years	11	24.4	100.0	36.4	[8.0; 64.8]				
≥70 years	11	24.4	100.0	0.0	na$				
**Systemic treatment**						0.618			
Dacarbazine	11	24.4	100.0	9.1	[0.0; 26.2]				
Temozolomide	34	75.6	94.1	21.8	[7.7; 35.9]				
**LDH**						0.007			
Elevated	14	34.1	92.9	0.0	na$		2.3	[1.2; 4.9]	0.014
Normal	27	65.9	96.3	22.2	[6.5; 37.9]		1.0		
Missing	4								

# 95%-CI = 95% confidence interval;

$ na = not available.

*4 patients had unknown values for LDH and were excluded; no confounding effects were detected.

Within both groups LDH remained the only independent factor according to Cox regression analysis and the relative risk do die from disease was still 2.3-fold increased in case of elevated LDH (p = 0.014 and p<0.001 for patients with or without brain metastasis, respectively). The slight trend for a better survival of patients treated by dacarbazine compared to temozolomide, which was observed in the entire cohort (p = 0.146), was completely lost in these additional analyses performed separately for both groups of patients.

## Discussion

In the present study, we did not observe any difference in survival after start of first-line chemotherapy according to the *BRAF* mutational status. All institutional patients, who received standard chemotherapy between the year 2000 and 2010 before availability of *BRAF* inhibitors were included without further selection. This is reflected by a high proportion of patients with brain metastases (21%) or elevated LDH (32%). Up to our best knowledge this is the first prognostic study in which mutational testing was performed retrospectively within the scope of this analysis and was not built upon already available data acquired from routine testing in therapeutic intention. Similar results were reported by Edlundh-Rose *et al.* who analyzed survival in 215 metastasized melanoma patients with available follow-up data in her study of 294 melanoma tumors from a total of 219 patients [12]. In contrast, other prior studies observed worse survival of patients with distant metastasis and *BRAF-V600* mutant melanoma [7]–[9], [11]. Worse prognosis was explained by the constitutive activation of the MAPK signaling pathway resulting in a more dynamic growth pattern of tumor cells but confounding effects due to patient selection could not be excluded in these studies [1], [2].

Conflicting results about prognostic impact of *BRAF-V600* tumor mutations are most likely due to patient selection and bias. In prior prognostic studies, data about the mutational status were mainly acquired due to intention to treat with a *BRAF- or MEK* inhibitor. But to analyze the treatment-unrelated “natural” impact of *BRAF-V600* tumor mutations, only patients with confirmed *BRAF*-mutations who finally did not receive subsequent inhibitor treatment can be considered. Reasons for non-treatment with inhibitors in *BRAF*-V600 mutant patients comprise applying of exclusion criteria in the frame of clinical studies (e.g. elevated LDH or development of brain metastases in the baseline imaging), decrease of performance status or early death due to disease progression. Therefore these patients represent a strongly biased cohort towards worse prognosis.

Moreover, these *BRAF*-mutant patients included in prior prognostic studies were compared to *BRAF* wild-type patients, which in turn were biased towards favorable prognosis. The analyzed cohorts comprised a large proportion of patients who were tested in the frame of clinical trials in which patients with cerebral metastases and high LDH levels were often excluded.

The only decisive factors for prognosis according to our analysis were LDH and the kind of visceral involvement. Cerebral involvement was more powerful than the visceral involvement according to AJCC to predict prognosis as described by others [20].

**Figure 2:**
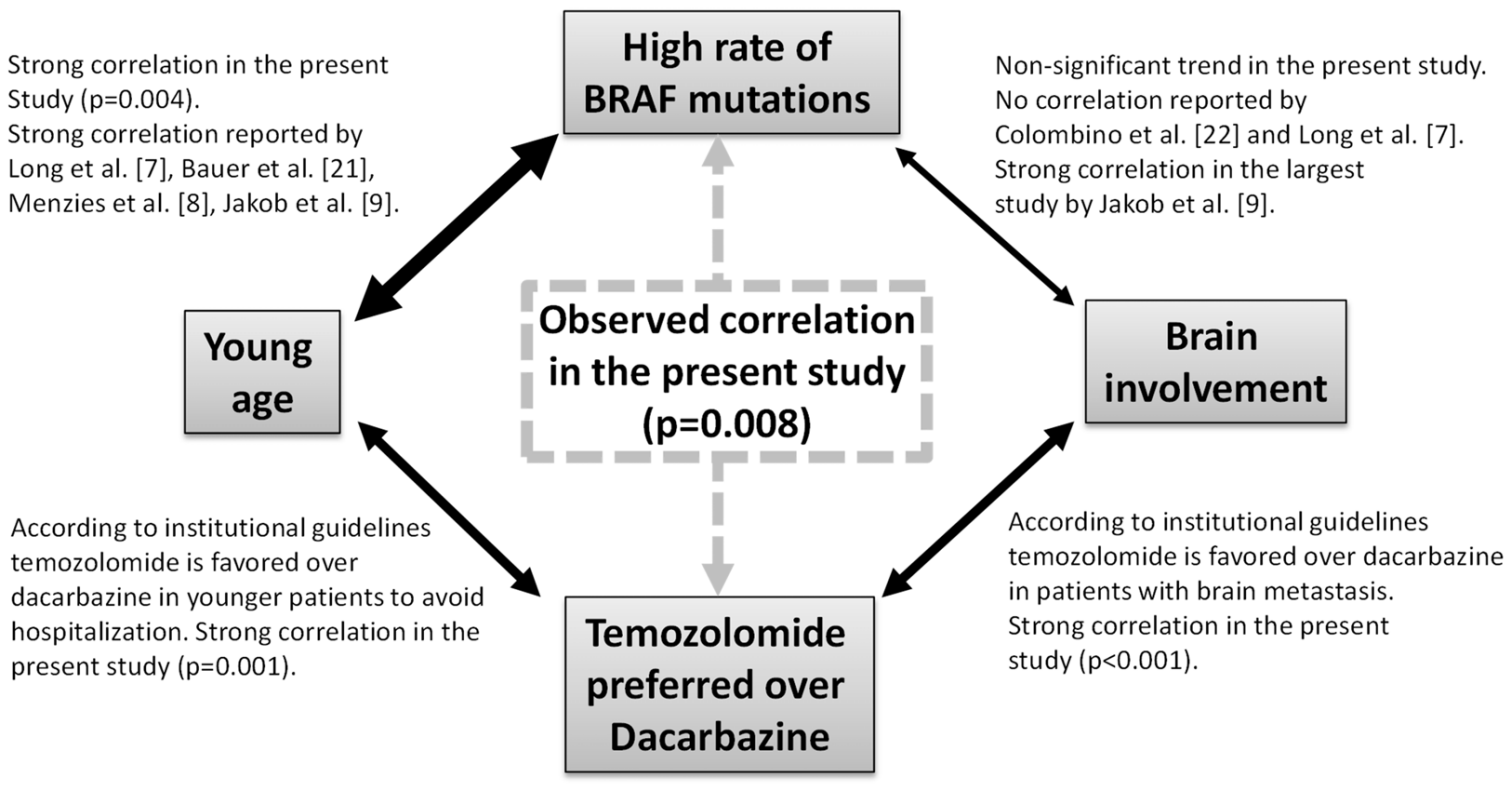
Correlation between a high rate of *BRAF V600* mutations and treatment with temozolomide. This unexpected correlation observed in the present study (grey broken arrow) can be explained as an indirect consequence of the appliance of institutional guidelines for treatment selection and established correlations (black arrows) between the rate of *BRAF V600* mutations or other clinical features (grey rectangles) reported in the literature and/or observed in the present study. The thickness of arrows illustrates the level of evidence for the given correlation.

In our study we found several correlations between analyzed factors which have to be discussed in more detail. The strong correlation between the treatment schedule and the *BRAF* status (p = 0.008) can be explained considering the following aspects: According to institutional guidelines, temozolomide is favored over dacarbazine in younger patients to avoid hospitalization for intravenous therapy and in those with brain metastasis. The appliance of these guidelines is reflected in our cohort by strong correlations between the selection of temozolomide and younger age (p = 0.001) or brain involvement (p<0.001), respectively. On the other hand both patient characteristics are in turn correlated with a high rate of *BRAF V600* mutations. A higher rate of mutant *BRAF* in younger patients was already reported in all larger studies of the last 5 years [7]–[9], [21] and this correlation was also observed in our cohort (Table 1; p = 0.004). The association between a high rate of *BRAF V600* mutations and a high prevalence of brain metastases is more controversial. A slightly higher rate of mutant *BRAF* was observed in our patients with brain metastases compared to those without (48.9% vs. 39.4%) but the difference was statistically not significant. In literature, a strong correlation was reported in the largest study, which analyzed this aspect thus far [9] in contrast to two prior studies [7], [22]. Nevertheless, the observed correlation between the selected treatment and the rate of *BRAF V600* mutations in the current study is therefore most likely an indirect consequence of appliance of institutional guidelines, which prefer temozolomide over dacarbazine in younger patients and in patients with brain metastases (Figure 2).

The trend (p = 0.145) for a worse survival of patients treated with temozolomide is also explained by the appliance of these guidelines. In our cohort, 47.2% temozolomide treated patients but only 7.7% dacarbazine treated patients had cerebral involvement. If patients with brain metastases are excluded the difference in survival according to the used agent is no longer observed (p = 0.962). Moreover, no impact of the BRAF status on survival was found in patients with (p = 0.575) or without (p = 0.714) brain metastases if analyzed separately according to cerebral involvement.

In conclusion, survival of melanoma patients receiving first line treatment with either dacarbazine or temozolomide is associated with the serum LDH level and cerebral involvement but not dependent on the tumor *BRAF-V600* mutational status.
